# Cell adhesion to agrin presented as a nanopatterned substrate is consistent with an interaction with the extracellular matrix and not transmembrane adhesion molecules

**DOI:** 10.1186/1471-2121-9-64

**Published:** 2008-12-04

**Authors:** Tobias Wolfram, Joachim P Spatz, Robert W Burgess

**Affiliations:** 1 Dept. New Materials and Biosystems, Max-Planck-Institute for Metals Research, University of Heidelberg, Stuttgart, Germany; 2 Dept. of Biophysical Chemistry, University of Heidelberg, Stuttgart, Germany; 3 Institute for Molecular Biophysics, Bar Harbor, ME, USA; 4 The Jackson Laboratory, Bar Harbor, ME, USA

## Abstract

**Background:**

Molecular spacing is important for cell adhesion in a number of ways, ranging from the ordered arrangement of matrix polymers extracellularly, to steric hindrance of adhesion/signaling complexes intracellularly. This has been demonstrated using nanopatterned RGD peptides, a canonical extracellular matrix ligand for integrin interactions. Cell adhesion was greatly reduced when the RGD-coated nanoparticles were separated by more than 60 nm, indicating a sharp spacing-dependent threshold for this form of cell adhesion.

**Results:**

Here we show a similar dependence of cell adhesion on the spacing of agrin, a protein that exists as both a secreted, matrix-bound form and a type-2 transmembrane form *in vivo*. Agrin was presented as a substrate for cell adhesion assays by anchoring recombinant protein to gold nanoparticles that were arrayed at tunable distances onto glass coverslips. Cells adhered well to nanopatterned agrin, and when presented as uniformly coated substrates, adhesion to agrin was comparable to other well-studied adhesion molecules, including N-Cadherin. Adhesion of both mouse primary cortical neurons and rat B35 neuroblastoma cells showed a spacing-dependent threshold, with a sharp drop in adhesion when the space between agrin-coated nanoparticles increased from 60 to 90 nm. In contrast, adhesion to N-Cadherin decreased gradually over the entire range of distances tested (uniform, 30, 60, 90, and 160 nm). The spacing of the agrin nanopattern also influenced cell motility, and peptide competition suggested adhesion was partially integrin dependent. Finally, differences in cell adhesion to C-terminal agrin fragments of different lengths were detected using nanopatterned substrates, and these differences were not evident using uniformly coated substrates.

**Conclusion:**

These results suggest nanopatterned substrates may provide a physiological presentation of adhesive substrates, and are consistent with cells adhering to agrin through a mechanism that more closely resembles an interaction with the extracellular matrix than a transmembrane adhesion molecule.

## Background

Proper cell adhesion is an important determinant for proliferation, migration and differentiation. Adhesion is often between ligands present in the extracellular matrix (ECM) and cell-surface proteins such as integrins, providing cells an anchor point for morphogenetic changes. Adhesion may also involve direct cell-cell contact via other transmembrane or cell surface-associated proteins. Cell-cell adhesion is particularly important in the nervous system, where there is a paucity of laminin/collagen rich matrix, but many sites of functional cell-cell interaction that regulate cell migration, axon pathfinding, and synapse formation and plasticity[1,2].

The proteoglycan agrin presents an interesting molecule for studies of cell adhesion, particularly for neurons. Agrin functions in neurodevelopment, and is an essential signal for maintaining postsynaptic differentiation at the neuromuscular junction (NMJ) [3]. Agrin exists in multiple protein isoforms, including a secreted, matrix-bound form that is found at the NMJ and in other basement membranes throughout the body, and a type 2 transmembrane form that is the predominant form expressed by neurons of the central nervous system [4-6]. Thus, it can be either a matrix-associated molecule, or a transmembrane, cell-associated molecule. It was our goal in these studies to determine if cells, and especially neurons, respond to agrin as they do to ECM ligands such as RGD peptides, or as they do to transmembrane adhesion molecules such as cadherins.

In assays using recombinant agrin as an adhesive substrate, the carboxy terminus of agrin was shown to mediate neuronal adhesion. Some, but not all, of this adhesion was dependent on Beta1 integrins, though a direct interaction of agrin and integrins was not established [5]. Thus, agrin may function as an ECM ligand for cell adhesion, similar to the laminins, with which it binds. Alternatively, the expression of the transmembrane form of agrin and structural similarities with other transmembrane adhesion molecules, such as neurexins, raise the possibility that agrin also functions as a transmembrane adhesion molecule in the nervous system [7]. The present study uses nanopatterned surfaces to examine the properties of cellular adhesion to molecularly defined agrin substrates, and compares these to adhesion mediated by cadherins, a well-established class of transmembrane, homophilic-adhesion molecules, and to previous work on RGD peptides, the canonical ECM ligand for integrin mediated adhesion [8-10].

Nanopatterned substrates offer a number of advantages for such studies. Gold nanoparticles can be deposited onto slides and biofunctionalized with the protein of interest, and the intervening space can be passivated with polyethylene glycol (PEG) to prevent non-specific interactions of the cell and substrate. Using this process, the stoichiometry, orientation, and spacing of the protein being presented as the adhesive substrate can be controlled. This approach has been used to study the adhesion, spreading, and motility of cells when presented with patterned RGD peptides arrayed on 5 nm spots that should only interact with single integrin complexes. This revealed a maximal spacing between spots of 58 nm before integrin mediated functions including adhesion, proliferation, and cell spreading, decreased sharply [8-10].

The results of the present study indicate that the adhesion profile of cells to patterned agrin more closely resembles that of RGD peptides than cadherins, even for neuroblastoma cell lines and primary cortical neurons. This suggests that cells recognize and adhere to agrin by mechanisms that are more closely related to the mechanisms of adhesion to the ECM than to transmembrane-protein-mediated cell/cell adhesion.

## Results

### Adhesion of cells to nanopatterned agrin

**Figure 1:**
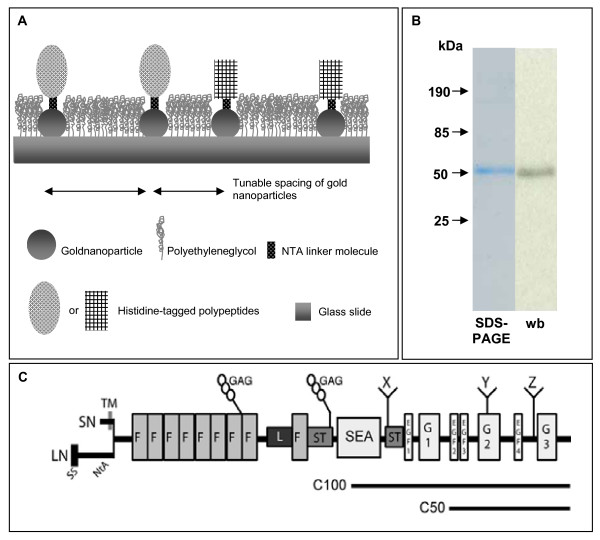
**Diagram of experimental system (A).** Gold nanoparticles were deposited onto glass with spacing determined by the coating process. The nanoparticles are functionalized with different constructs of recombinant Agrin (or N-Cadherins) via NTA-histidine interaction. The biofunctionalization and spacing between dots on the nanometer scale was varied, and the dependence of cellular behaviour on these parameters was determined. B) Purification of Agrin analyzed by SDS-PAGE analysis and western blotting. C) Two carboxy-terminal agrin constructs (C100 and C50) were used in this study (SN – Short transmembrane N-terminus, LN- long, secreted N-terminus, F – follistatin repeat, GAG – glycosaminoglycan domain, SEA – Sea Urchin Sperm protein/enterokinase/agrin domain, ST – seronine/threonine rich region, EGF – EGF like repeat, G – laminin like globular domain, X, Y, Z sites of alternative splicing).

In this study, agrin was used as a nanopatterned substrate for cell adhesion assays. With this technique, gold nanoparticles 5–8 nm in diameter are arrayed on a glass slide at tunable distances ranging from 30 to 160 nm between particles (Figure 1A). The intervening glass surface is passivated with polyethylene glycol (PEG) to prevent adhesion to areas other than the functionalized nanoparticles. A construct consisting of the carboxy-terminal quarter (50 kDa) of rat agrin with C-terminal Myc and 6X-histidine tags was expressed in Cos-7 cells and purified from the media by nickel chromatography (C50) (Figure 1B). In addition, a commercial agrin construct consisting of the C-terminal half of agrin (100 kDa), with an N-terminal histidine tag, was also used (C100) (Figure 1C). The nanoparticles were functionalized with these constructs using a thiol-NTA linkage that reacts with the gold in the substrate and the nickel bound by the histidine tag. Using this approach, agrin is presented as a substrate for cell adhesion with a controlled spacing, limited stoichiometry, and fixed orientation. The validation and characterization of agrin binding to the gold nanoparticles is described in detail elsewhere [11-13].

**Figure 2:**
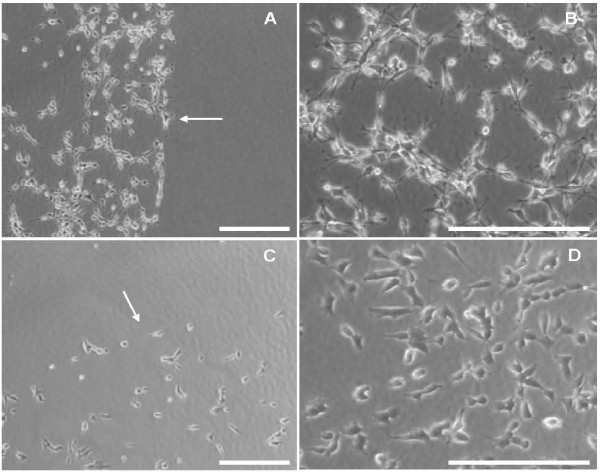
**Rat B35 neuroblastoma cells (A and B) and human SHSY-5Y neuroblastoma cells (C and D) on C100-Agrin biofunctionalized 60 nm substrates.** Depicted is in A and C the dipping edge (arrows) and an overview of the cell population on biofunctionalized gold particles. Both cell types are able to build short cellular protrusions and the overall cell morphology is normal. Scale bars = 200 μm.

Agrin is known to be a permissive substrate for cell adhesion when presented in a uniform coating. The ability of agrin to mediate cell adhesion when presented as a nanopatterned substrate was tested using neuroblastoma cell lines and agrin spaced at 60 nm. Cells were incubated on the substrates for four hours, non-adherent cells were washed off, and bound cells were quantified using a colorimetric assay (see Methods). Both rat B35 cells and human SHSY-5Y cells bound specifically to the agrin substrate (Figure 2). In this and subsequent experiments, both C50 and C100 agrin constructs were used with similar results. The data shown is for C100 agrin unless otherwise noted. No cell adhesion was seen on the PEG-passivated portions of the slide that did not also have agrin functionalized gold nanoparticles. Adherent cells showed normal morphology, with a slightly elongated soma and short processes. The "dipping edge" is also seen in figure 2A, C (Arrows). This is a region of artifactually high particle density that forms when the nanopatterned surfaces are made using the dip-coating procedure (see Methods). Therefore, cells efficiently adhere to agrin when presented as a nanopatterned surface with 60 nm spacing.

### Comparison of adhesive substrates

**Figure 3:**
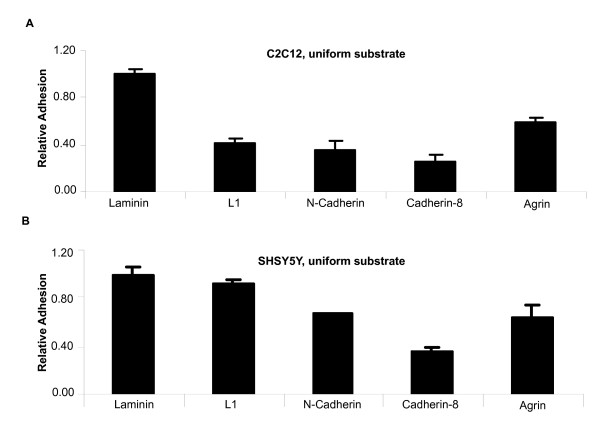
**Static cell adhesion assays on uniformly coated substrates.** Different cell-cell adhesion molecules were used to evaluate cell adhesion of C2C12 mouse myoblasts (A) and SHSY-5Y human neuroblastoma cells (B). Values for adhesion to Laminin, a well-known component of cell matrix, were set to one relative unit. Agrin C100 functions as an adhesion molecule within the same range as Laminin, Cadherins, and Immunoglobulin superfamily members. Values are averages of three independent trial ± S.D.

The goal of this study was to determine if adhesion to agrin was more characteristic of adhesion to extracellular matrix components, or to a cell surface adhesion molecule. We therefore first compared agrin to other classes of adhesion molecules using uniformly coated surfaces. These other adhesive substrates included 1) laminin, an extracellular matrix component, 2) L1, a Ig-superfamily member transmembrane adhesion molecule, and 3,4) N-cadherin and cadherin8, two classic homophilic cell surface adhesion molecules. Both mouse C2C12 myoblasts and human SHSY5Y neuroblastoma cells were tested with similar results (Figure 3). C2C12 cells bound all substrates significantly less efficiently than laminin (p values = 0.0002). However, adhesion to agrin was significantly higher than adhesion to N-Cadherin or Cadherin8 (p = 0.004 and 0.0003 respectively). SHSY5Y cells bound agrin and L1 without a significant reduction compared to laminin. Binding to N-Cadherin and Cadherin8 was significantly reduced relative to laminin (p = 0.0007 and 0.0002 respectively), but only Cadherin8 differed significantly from agrin (p = 0.01). Three independent adhesion assays were performed for each substrate and cell type, and adhesion was standardized to the amount of adhesion observed for laminin in each trial. Background binding on BSA coated substrates was subtracted and all substrates were deposited using 10 μg/ml solutions of recombinant protein.

**Figure 4:**
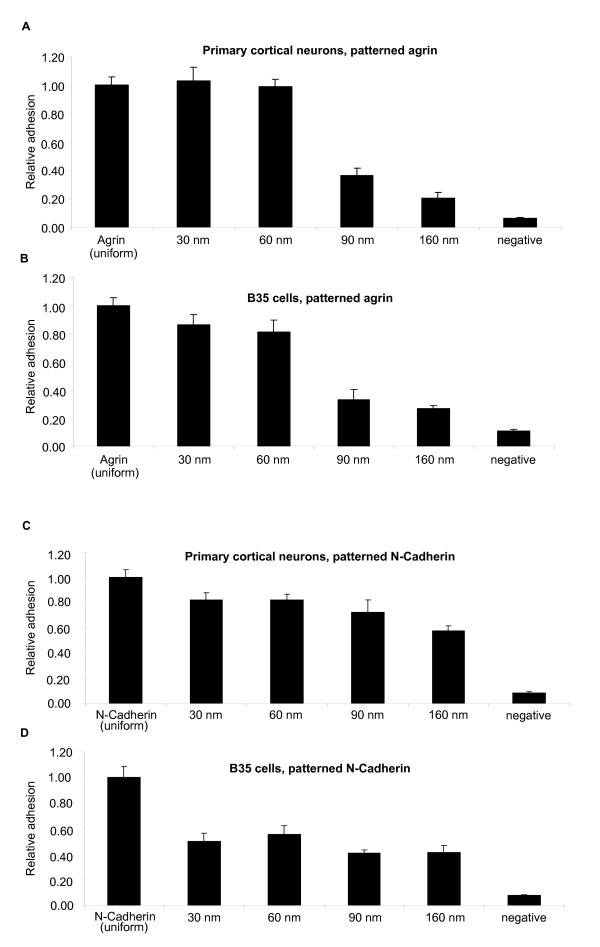
**Cell adhesion on C100 Agrin nanopatterned substrates.** Mouse primary cortical neurons (A) showed significantly higher adhesion to 30 nm and 60 nm spaced substrates than to 90 nm and 160 nm spaced substrates. Adhesion to more closely spaced substrates is comparable to homogenously coated agrin (first bar). Rat B35 neuroblastoma cells (B) showed a similar behaviour. Cell Adhesion on N-Cadherin nanopatterned substrates. Primary mouse cortical neurons (C) and Rat B35 neuroblastoma cells (D) do not show a significant decrease in cell adhesion with increasing spacing of the nanopatterned substrates, and adhesion is always below the level observed for uniformly coated N-Cadherin (first bar). This suggests a different mechanism for N-Cadherin mediated cell adhesion than for Agrin mediated cell adhesion.

Agrin and N-Cadherin were next tested for their dependence on spacing of the nanopattern. This provided a comparison of agrin to a homophilic adhesion molecule where both provided good adhesion for a neuroblastoma cell line. In each case, both primary mouse cortical neurons and rat B35 neuroblastoma cells were tested (Figure 4). As above, results were obtained for three independent assays and adhesion was standardized to uniformly coated agrin or N-cadherin. For agrin, adhesion of both primary cortical neurons and B35 cells was comparable to uniformly coated protein at spacings of 30 and 60 nm (Figure 4A, B). Primary cortical neurons did show a small but significant reduction in adhesion to 60 nm spaced agrin (p = 0.004), but the difference was not significant compared to 30 nm spaced agrin (p = 0.8). However, adhesion dropped significantly with 90 and 160 nm spacing (p values = 0.0002). Adhesion to the 160 nm spaced agrin was comparable to the negative control for both cell types (p = 0.02 and 0.03). Negative controls were created using BSA coated gold nanoparticles in the same PEG passivated environment.

The decreased agrin adhesion seen with increased spacing may be an under-estimate. In more closely spaced patterns, the cells were clearly adhering to the surface and had normal morphologies. At 90 and 160 nm, the cells were clumped, adhering to one another if one cell got purchase on the substrate. This was true of both B35 cells and primary neurons, with the primary cells clumping more quickly than the neuroblastoma cells. Quantification of this effect for the B35 cells is shown in Additional file 1. This effect is probably mediated by classical cell-cell adhesion mechanisms, although disrupting these mechanisms through approaches such as the chelation of divalent cations decreases adhesion overall [5], making this assumption difficult to test. Thus, as the values reported are based on the number of adherent cells/sample, the number of cells preferentially binding the substrate, as opposed to binding other cells, may be even lower.

Unlike nanopatterned agrin, cell adhesion to N-cadherin showed no sharp spacing-dependent threshold (Figure 4C, D). For both cell types (primary cortical neurons and B35 cells), the adhesion to patterned substrates was significantly lower than to uniformly coated N-cadherin (p values from 0.005 to 0.0002). The amount of adhesion decreased gradually as the spacing of the nanopattern increased. No single step (30 to 60, 60 to 90, 90 to 160 nm) produced a significant decrease in adhesion, but all patterned N-cadherin substrates showed adhesion significantly above the negative control (p = 0.0002). Like cells on patterned agrin, cell clumping was also observed on the more widely spaced N-cadherin patterns. This again suggests that the amount of adhesion on widely spaced N-cadherin substrates may be an over-estimate, but unlike agrin, these values were still significantly higher than negative controls. Therefore, adhesion to N-Cadherin is dependent on spacing, but the relationship varies more linearly with spacing and does not show a critical threshold as agrin-mediated adhesion did.

### Cell motility on nanopatterned agrin

**Figure 5:**
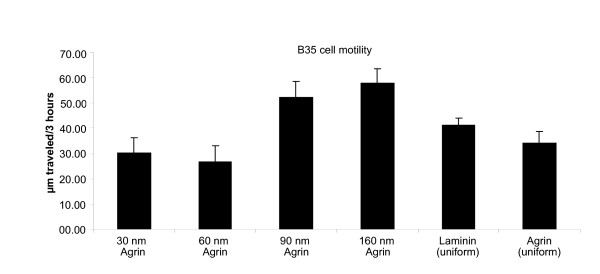
**Cell motility rates of B35 cells on different agrin C100 nanopatterned surfaces.** Single cells were evaluated for 3 hours with live cell imaging with DIC optics. Cells appear to have lower motility rates on more closely spaced agrin C100 substrates. Interestingly, homogenously coated substrates show intermediate values.

The spacing of the nanopatterned agrin also influenced cell motility (Figure 5), and this inversely correlated with adhesion. Cells on more closely spaced patterns (30 and 60 nm) displayed less mobility than cells on more widely spaced patterns (90, 160 nm). Interestingly, the mobility differences were not purely a function of the cells' ability to adhere, as cells on uniformly coated agrin or laminin had intermediate motilities.

Motile cells often extend a leading process during migration; however, cell motility also inversely correlated with the extension of processes, as assessed by a cell-spreading index (see Additional file 2). On more closely spaced agrin patterns, cells were flatter and extended short protrusions. On more widely spaced agrin patterns, cells extended fewer processes, had rounder cell bodies, and occupied less surface area. Thus, strong cell adhesion to closely spaced patterns led to the anticipated effects on cell morphology and spreading, but the looser adhesion of widely spaced patterns was more conducive to increased cell motility.

### Molecular mechanisms

**Figure 6:**
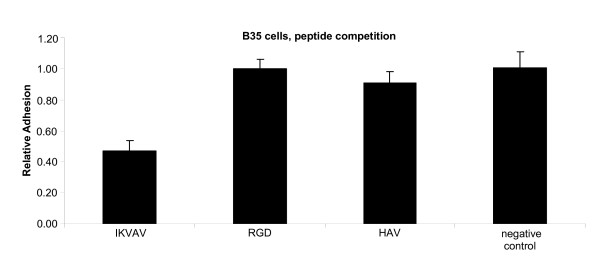
**Peptide inhibition assay on 60 nm spaced C100 Agrin substrates.** The IKVAV peptide reduces B35 cell adhesion to approximately 50% of the control (no peptide included). RGD and HAV do not significantly affect cell adhesion.

The adhesion profile of cells on agrin nanopatterned arrays (Figure 4) is very similar to the profile previously reported for cells adhering to patterned RGD peptides, where adhesion also dropped at > 60 nm spacing [8]. Since RGD is the canonical extracellular matrix ligand for integrin-mediated cell adhesion [14], we tested the possible involvement of integrin signaling in adhesion to agrin by adding competing peptides to cell adhesion assays with 60 nm agrin spacing (Figure 6). The peptide IKVAV (an active peptide found in the laminin alpha1 chain) significantly reduced adhesion compared to control assays performed with no competing peptide (p = 0.0002). This effect was specific, and no decrease in adhesion was seen using RGD or HAV (found in N-Cadherin) peptides compared to the controls.

**Figure 7:**
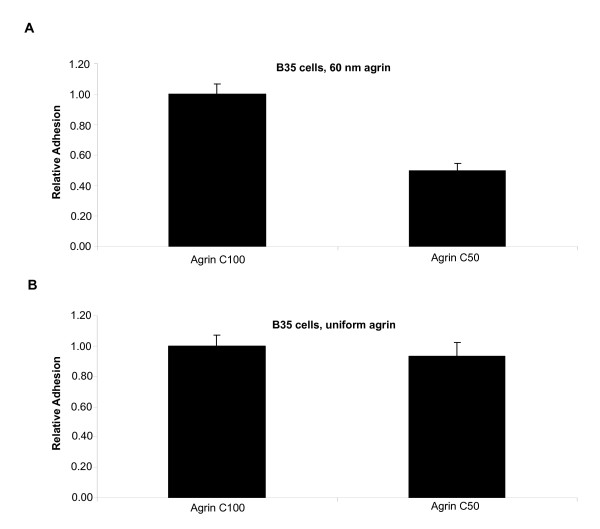
**Different B35 cell adhesion behaviour with different presentation of the Agrin C-terminal constructs.** (A). On agrin substrates spaced at 60 nm, the C50 and C100 proteins show a difference in cell adhesion. B) This effect is not seen using homogenously coated substrates, where cells adhere equally well to both constructs.

We also tested whether presenting agrin as a patterned versus unpatterned substrate influenced adhesion (Figure 7). Indeed differences in cell adhesion were seen in comparing the C-terminal 100 versus 50 kDa fragments when each was patterned with 60 nm spacing, with C50 conferring approximately half as much adhesion as C100 (p = 0.00002, ttest) (Figure 7A). However, consistent with previous studies, no differences were seen when these two fragments were coated homogeneously (p = 0.6, ttest) (Figure 7B). There are two possible explanations for this difference. First, a mechanism for adhesion to C50 may be functional when it is presented uniformly, but lost when it is presented as a patterned substrate. However, this mechanism would need to be retained in the C100 fragment, even when it is nanopatterned. Alternatively, the C50 and C100 constructs are presented in the reverse orientation by virtue of their respective C-terminal and N-terminal 6X His tags in the thiol-NTA functionalization of the gold nanoparticles; whereas, they are presented in a random orientation when homogeneously coated. In either case, the differences detected by the patterned presentation further suggest the usefulness of this approach for assessing cellular responses to conditions that can be controlled at the molecular scale.

## Discussion

We have shown that agrin presented as a nanopatterned substrate mediates adhesion for a variety of cell types, including primary neurons and neuroblastoma cell lines. In addition, we have shown that the dependence of adhesion on the spacing of the agrin nanopattern has a sharp spacing-dependent threshold, similar to that of the canonical integrin ligand RGD, a component of many ECM proteins [14]. In contrast, the cell-surface homophilic adhesion molecule N-Cadherin has a more linear dependence on spacing, with no significant spacing-dependent threshold. Nanopatterned spacing also influences cell motility and morphology, but not simply in parallel with adhesion. Consistent with cells responding to agrin as they would to integrin-dependent extracellular matrix ligands, adhesion was inhibited by competing IKVAV peptides. Finally, nanopatterned substrates are capable of highlighting differing cell responses that are masked when the substrate is uniformly presented.

In addition to the effects on cell adhesion that we show, cell proliferation had a similar dependence on the spacing of agrin substrates (not shown). Both proliferation and adhesion are often integrin-mediated processes, and the inhibition of adhesion by the IKVAV peptide is consistent with this. The IKVAV sequence was identified as an active motif in the laminin alpha1 chain that promoted cell adhesion, proliferation, and neurite extension, and these effects depend, at least in part, on Beta1 integrins [15-17]. As such, the RGD and IKVAV peptides have been previously reported to be similarly effective [15]. Agrin contains neither an RGD nor an IKVAV sequence; however, previous studies have shown that adhesion of chick primary ciliary ganglion neurons to agrin was largely dependent on Beta1 integrins, and was sensitive to RGD peptide competition at a site that was also sensitive to function blocking αV integrin antibodies. A second, non-RGD-sensitive site of integrin-dependent cell-adhesion mapped to a more C-terminal portion of agrin, although both sites are contained within the C50 fragment [5]. The insensitivity of rat B35 neuroblastoma cells to RGD peptide competition in this study is interesting and may reflect a greater dependence in this cell type for adhesion to the more C-terminal domain of agrin. Together, these results strongly suggest that adhesion to agrin is at least in part integrin dependent, and the absence of an IKVAV or RGD peptide may indicate that the interaction is either indirect or mediated by a less-well defined domain. Despite the lack of primary sequence homology, agrin and laminins do share many structural similarities, including EGF-like repeats and laminin-type globular domains, found in the C-terminus of agrin.

The N-terminus of agrin and its heparan sulfate additions have been extensively studied for their role in stopping neurite outgrowth and promoting nerve terminal differentiation [18,19]. Agrin has also been used in other biophysical studies in which its presentation was spatially restricted either by using microcontact printing to pattern substrates [20], or microfluidics to restrict the point of agrin application [21,22]. These studies were designed to investigate the spatial properties of agrin signaling in synaptogenesis on muscle cells, an activity that maps to the C-terminal portion of the protein, which is not subject to glycosaminoglycan addition.

Since agrin exists as both a matrix protein and a transmembrane protein in vivo, we felt it was particularly important to determine in which context cells are responding to it [5,6]. The extracellular matrix bound isoform is the active form for neuromuscular junction synaptogenesis [4]. However, agrin's role in the central nervous system, where the transmembrane isoform predominates, remains less clear. The loss of agrin effects synapse density in the brain, and through interactions with the K-Na-ATPase, is proposed to mediate activity dependent plasticity [23,24]. Agrin also activates both C-Fos and CREB signaling in neurons [25,26]. Given these disparate effects, it is difficult to know what *in vitro *assays may most accurately measure agrin's true function. We chose cell adhesion as a first attempt to examine how neurons respond to agrin, in part because this assay also allowed the comparison to well-known components of the extracellular matrix and to well-established transmembrane adhesion molecules, such as L1 and cadherins. It is interesting that even neurons respond to agrin more as cells respond to the ECM ligand RGD than as they do to the transmembrane proteins N-cadherin.

Why is there a sharp threshold for adhesion to agrin and RGD, but not N-cadherin? First, we have to acknowledge that the comparison of agrin to one integrin-dependent matrix ligand and one homophilic transmembrane adhesion molecule is not exhaustive, but the consistencies and inconsistencies are noteworthy. Our preferred explanation is that adhesion to agrin requires an intracellular signaling cascade, and the formation of an adhesion complex analogous (if not identical) to that of integrin-mediated adhesion to RGD. The size of these complexes inside the cell may create a lower limit for spacing; presumably their effectiveness is reduced if the sites of adhesion are spaced more closely than the diameter of such a complex. This could explain the saturating effect seen at 30 and 60 nm spacing, which were similar to uniform coating. The threshold for decreasing adhesion would arise when the avidity of these complexes is no longer sufficient to cause adhesion, not through the lack of signal at an individual complex, but from the lack of sufficient cumulative signal from the array of adhesive sites. In contrast, the N-cadherin mediated adhesion may be more analogous to mechanical "Stickiness" at the cell surface, where the force required to disrupt adhesion varies more linearly with the protein's density on the membrane. The absence of an intracellular cascade would eliminate the threshold, and as long as some of the protein is present there will be some amount of adhesion, with the upper limit being defined at the molecular scale and by the affinity of the interaction.

The affinity of the cellular receptors for the ligand may also contribute to the agrin threshold effect. If this is the case, it is interesting to note that integrins have a much lower affinity for RGD peptides than for full-length fibronectin [27], yet large agrin fragments have an apparently similar threshold to RGD peptide. However, in considering affinity as an explanation, it is also important to consider the constraints of our system. Each gold particle is bound with only one or sometimes two proteins, and this number should not change as the spacing changes [13]. Therefore, at the molecular level, the number of ligands presented to a cell-surface receptor is not changed; it is the spacing between these sites of interaction this changes. This effectively decreases the density of potential attachment sites seen by a single cell by the square of the change in spacing, but does not change the molecular composition of an individual attachment site. Therefore, avidity, and not classic receptor-ligand binding affinity, is the variable. A biophysical test of this would be to measure the force of cell adhesion as the spacing of agrin and N-cadherin is increased to confirm that our measures of adhesion co-vary with mechanical force.

The subtleties of cellular responses to agrin, such as the orientation of the protein, may have further implications for function. For instance, the anti-parallel presentation created by the N-terminal 6X His tag of C100 may more closely resemble membrane-to-membrane interactions or suggest an orientation of agrin within the meshwork of matrix that is optimal for cellular recognition and binding.

In considering molecular spacing, laminin polymers in a self-assembling meshwork form a polygon pattern with vertices separated by 30–40 nanometers, comparable to the 30–60 nanometer spacing of agrin-coated nanoparticles that conferred optimal cell adhesion. Laminin polymerization also organizes other cell-surface proteins, including dystroglycan, another receptor interacting with the C-terminus of agrin [28-31]. Thus our results appear to be in a physiologically relevant range of distances and the patterning of ECM-associated ligands with such spacing may actually provide a more physiological presentation. However, there are also limitations to the use of nanopatterned substrates. For instance, if cell adhesion depends on clustered adhesion molecules, the size of the gold-nanoparticles as well as their spacing would be expected to have a strong influence. Also, if adhesion depends on a mix of proteins, either as homo-dimers or in multiprotein complexes, the stoichiometric presentation of the substrates would be more difficult to control.

An additional distinction between uniformly coated substrates and the nanopatterned substrates such as we have described is the thickness and rigidity of the substrate itself. Homogeneous coating of glass with recombinant proteins applied at 10 μg/ml results in a film of protein that can be 100–200 nm thick (T. W. unpublished observations). However, molecules directly anchored to the nanoparticles are presented in comparatively low copy number and the thickness of the surrounding PEG-passivation can be controlled by polymer length. Therefore, the nanopatterned substrates are presumably more rigid and more reproducible in their thickness and stiffness than homogeneous substrates. Substrate rigidity has been shown to have a variety of effects on cell morphology, proliferation, and differentiation [32,33]. These effects may also be influencing the cellular response to homogeneous versus patterned substrates, particularly for parameters like cell motility.

## Conclusion

The results presented above are most consistent with cells adhering to agrin through mechanisms that more closely resemble interactions with the ECM and ligands such as RGD, than with transmembrane adhesion molecules such as N-Cadherin, which we tested in parallel. These findings are of particular significance for agrin, which exists as both a secreted, ECM-bound protein and as a type-2 transmembrane protein. We were most interested in examining the adhesion properties of neurons, because the transmembrane form of agrin is the predominant isoform found in the central nervous system, but its function there remains unclear. Therefore, it is interesting that neurons recognize agrin as though it was a matrix-presented ligand.

Future experiments can take advantage of the nanopatterned presentation of molecules such as agrin to address additional questions of cell biology. For example, if agrin is presented in the proper orientation with 30 nm spacing in a grid 250 nm across, does it induce pre- or postsynaptic specializations in cultured neurons? Such a focal, anchored presentation, which would approximate the point of contact between an outgrowing axon and its postsynaptic target, may cause very different effects than bath application of soluble agrin in the media. Such an experiment may bridge the apparent gap in agrin's central function in NMJ formation with its modest effects on synapse formation in the central nervous system. The results presented here not only establish the feasibility of such studies, they also provide mechanistic suggestions for how cells respond to agrin in comparison to other adhesion molecules.

## Methods

### Recombinant Agrin and Cadherins

The recombinant C-terminal half of agrin was purchased from R&D Systems (550-Ag) and includes amino acids 1153 to the end of the protein (1948), with a synthetic N-terminal signal peptide for secretion and a 6X-histidine tag for purification. This protein is herein referred to as C100 agrin (C-terminal 100 kDa fragment). An expression construct for the C-terminal quarter of agrin (C-terminal 50 kDa fragment, or C50) was generated using following oligonucleotides for amplification from mouse brain cDNA: 5'Agrin-BglII TAT A**AG ATC T**CC CTG CCA GCC GAA CCC CTG and 3'Agrin-XhoI ATA T**CT CGA G**AG TGG GGC AGG GTTC TTA G. Synthetic restriction enzyme sites are in bold. The amplification product (1434 bp of agrin) was cloned into the BglII and XhoI sites of the AP-5 Tag vector (Gene Hunter), which provides an in frame N-terminal signal peptide for secretion and a C-terminal myc-epitope tag and 6X histidine tag for purification. The sequence of the cloned PCR product was confirmed. This fragment is the equivalent of the previously reported human agrin fragment Δ 1480 [5].

Cadherin expression constructs were similarly prepared. N-cadherin sequences were amplified from mouse brain cDNA using the following primers: 5' ATAT**AGATCT**GGTGAAATTGCATTATGCAAG and 3' ATAT**CTCGAG**GCCCGTGCCAAGCCCTGCA. The amplified PCR products were cloned into Topo II vectors (Invitrogen), sequenced, and then cloned into the AP5 vector using BglII and XhoI (bold sites). Cadherin8 was amplified using 5' ATAT**GGCCCAGCCGGCC**GCTCCGATGAATCAGGCTCAC and 3' ATAT**GGGCCC**CATACTGAGTCCAATAGGAAGG, cloned into TopoI, sequenced, and moved into AP5 by SfiI and ApaI (bold) digestion and ligation. In all cases, expression of full-length protein products and purity were assessed by SDS-PAGE of recombinant protein purified by Nickel chromatography (Novagen). All constructs were expressed in COS-7 cells using Lipofectamine 2000 for the generation of recombinant protein.

### Generation of gold nanopatterned surfaces

Gold nanoparticle patterns from diblock copolymer micelles were prepared on glass cover slips (Roth, Germany) as previously described [8,34]. The molecular characteristics of polystyrene-block-poly(2-vinylpyridine) (PS-b-P2VP), diblock copolymer PS(500)-b-P2VP(270), and diblock copolymer PS(990)-b-(385) were described previously [8,11]. In brief, micellar suspensions are coated onto glass slides as monolayers. The organic components of the micelles are then removed by heating in a plasma oven and the gold nanoparticles are deposited onto the glass. The spacing of the gold nanoparticles is determined by the size of the organic compounds in the micelles. Gold-free regions of the glass were passivated to prevent unspecified protein attachment by immobilizing linear polyethylene glycol (PEG, CH3-(O-CH2-CH2)17-NH-CO-NH-CH2-CH2-CH2-Si(OEt)3) to the glass coverslips by first chemically activating the substrates (H_2_SO_4_/H_2_O_2 _= 3:1) followed by substrate immersion in 1 mM PEG solution in dry toluene (Merck, Germany) under nitrogen atmosphere and incubation in oil bath at 80°C for 24 hours [35]. Substrates were then rinsed extensively with methanol and ethyl acetate (Aldrich, USA), blown dry with nitrogen, and used directly for biofunctionalization. Nanodot patterns were created with interspacings of 30 ± 4, 60 ± 8, 90 ± 11, and 160 ± 23 nm, and gold particle diameters of 5 to 8 nm. The qquality of the gold nanoparticle patterns was verified by scanning electron microscopy and atomic force microscopy.

### Biofunctionalization of nanopatterned surfaces

Mono-thiol NTA was used to link gold nanoparticles to the recombinant proteins. First, PEG-functionalized substrates were immersed for 4 h in 1 mM mono-thiol NTA in ethanol. Samples were washed with MilliQ-water, and briefly incubated with 10 mM Ni^2+ ^in HEPES buffered saline (HBS). Following washes with phosphate-buffered saline (PBS), the substrates were incubated with 1 μg/ml 6X-His tagged recombinant protein in PBS for the different protein domains of agrin or cadherins over night at 4°C. Biofunctionalization was verified by immunofluorescence staining and immunogold labeling as described previously [12,13]. Immunogold labeling showed single protein functionalization for most of the gold nanoparticles.

Uniformly coated substrates were prepared by covering O_2_-plasma activated cover slips with glutaraldehyde solution (2% in water) for 15 minutes at room temperature. After washing, protein solutions at 10 μg/ml in PBS were applied and incubated for 30 minutes before removing the excess solution and rinsing.

### Cell culture and adhesion assays

Neuroblastoma cells (B35 and SHSY5Y) were maintained in DMEM supplemented with 10% fetal bovine serum (FBS), and penicillin/streptomycin (PS) in a humidified atmosphere containing 5% CO_2 _at 37°C. Myoblasts (C2C12) were grown in DMEM supplemented with 20% FBS and PS in 10% CO_2 _at 37°C. Primary cortical cultures were prepared from dissociated neurons of embryonic day 15 C57BL/6J mouse embryos. Cortices were dissected free, dissociated by a 12 minute incubation in trypsin in HBSS, followed by tituration, and cultured in neuralbasal media supplemented with B27 supplement (GIBCO) (2% vol/vol), L-Glutamine (1%vol/vol), and penicillin/streptomycin (PS).

Static adhesion assays were performed as described previously [36,37]. In brief, for homogeneously coated substrates with Laminin, L1, N-Cadherin, Cadherin-8, and Agrin (all 10 μg/ml in PBS) substrates were incubated with 10^5 ^SHSY-5Y human neuroblastoma cells or C2C12 mouse myoblastoma cells. For nanopatterned Agrin or N-Cadherin substrates, 30, 60, 90, and 160 nm substrates were incubated with 10^5 ^B35 rat neuroblastoma cells or 10^6 ^primary cortical neurons per sample. Cells were cultured for 4 hours at 37°C in starvation medium (1% FBS) or neurobasal medium and rinsed three times with prewarmed PBS. To each sample 300 μl of substrate solution with 3.75 mM *p*-nitropheno-N-acetyl-β-D-glucosaminide (Sigma) in 50 mM citrate buffer was added and cells were incubated at 37°C for 30 minutes. After incubation the reaction was stopped by adding 450 μl of stop solution containing 5 mM ethylene diamine tetra-acetic acid (Sigma) in 50 mM glycine buffer. 150 μl of each sample were transferred into a 96 well plate (Falcon) and the absorbance at 405 nm was measured with a microplate reader (Tecan). Relative cell adhesion was measured by setting the homogeneously coated Agrin or N-Cadherin control to 1 for each assay. All assays involved triplicate trials with independently coated substrates, and the average of these was considered an n = 1. Each assay was repeated minimally three times, and significance was determined using One-Way Analysis of Variance (ANOVA) unless otherwise noted, with p ≤ 0.01 considered significant.

For adhesion assays with peptide-inhibition, cells were incubated with 100 μg/ml RGD peptide (Sigma), 100 μg/ml IKVAV peptide (Sigma), or 1 mg/ml HAV peptide (American Peptide). Cells were mixed in high-glucose DMEM with the peptides and incubated for 30 min at 4°C prior to plating on 60 nm spaced Agrin substrates. Cell adhesion was assayed as described above.

### Cell motility and cell spreading assays

Cells on their respective substrates were imaged for 3 hours using differential interference contrast (DIC) optics on a Leica SP2 microscope with environmentally controlled stage. Cells were maintained in starvation media with 1% FBS during these experiments. Images were obtained every 10 minutes, and motility rates were determined using ImageJ software. Three to five cells per field in 5 different samples per surface-type were analyzed. Cell motility was evaluated by analyzing the change in the location of the center of each cell at 10 min intervals.

The measurements of the cell area (cell surface) were analyzed after 6 hours incubation on each substrate. Cell boundaries were marked in ImageJ, analyzed and compared to the average of the cell surface area value obtained on homogenously coated Agrin and Laminin substrates

## Abbreviations

ECM: extracellular matrix; NMJ: neuromuscular junction; PEG: polyethylene glycol; C100: 100 kilodalton carboxy: terminal half of agrin; C50: 50 kilodalton carboxy: terminal quarter of agrin; BSA: bovine serum albumin; PBS: phosphate buffered saline; nm: nanometer; DMEM: Dulbecco's Modified Eagle's Medium; DIC: differential interference contrast; SDS: PAGE: sodium dodecyl sulfate polyacrylamide gel electrophoresis; kDa: kilodaltons; FBS: fetal bovine serum; ANOVA: analysis of variance; PS: polystyrene; cDNA: complementary DNA.

## Authors' contributions

TW acquired and analyzed the data; he also was critical for experimental design and for preparation of the manuscript. JPS provided essential technical expertise on the construction of gold nanopatterns and data interpretation, as well as critical input on preparation of the manuscript. RWB oversaw the experimental design and data interpretation and generated the agrin and N-Cadherin expression constructs used. He also was critical for the writing of the manuscript. All authors read and approved the manuscript.

## Supplementary Material

Additional file 1 **Cell clustering on nanopatterned Agrin functionalized substrates.** The tendency of cells to stick to one another rather than to widely spaced (90 and 160 nm) Agrin coated substrates is shown. Click here for file

Additional file 2 **Cell spreading on nanopatterned substrates.** Cells show greater spreading on closely spaced Agrin substrates (30, 60 nm), but have rounder morphologies with less surface area in contact with 90 and 160 nm substrates. Click here for file
