# Supplementary material for: Cell adhesion to agrin presented as a nanopatterned substrate is consistent with an interaction with the extracellular matrix and not transmembrane adhesion molecules
Source: BMC Cell Biol. 2008 Dec 4;9:64. doi: 10.1186/1471-2121-9-64 (PMC2612657; doi:10.1186/1471-2121-9-64)
Supplement: Additional file 1 — Cell clustering on nanopatterned Agrin functionalized substrates. The tendency of cells to stick to one another rather than to widely spaced (90 and 160 nm) Agrin coated substrates is shown. [file 1471-2121-9-64-S1.pdf]

Additional file 1

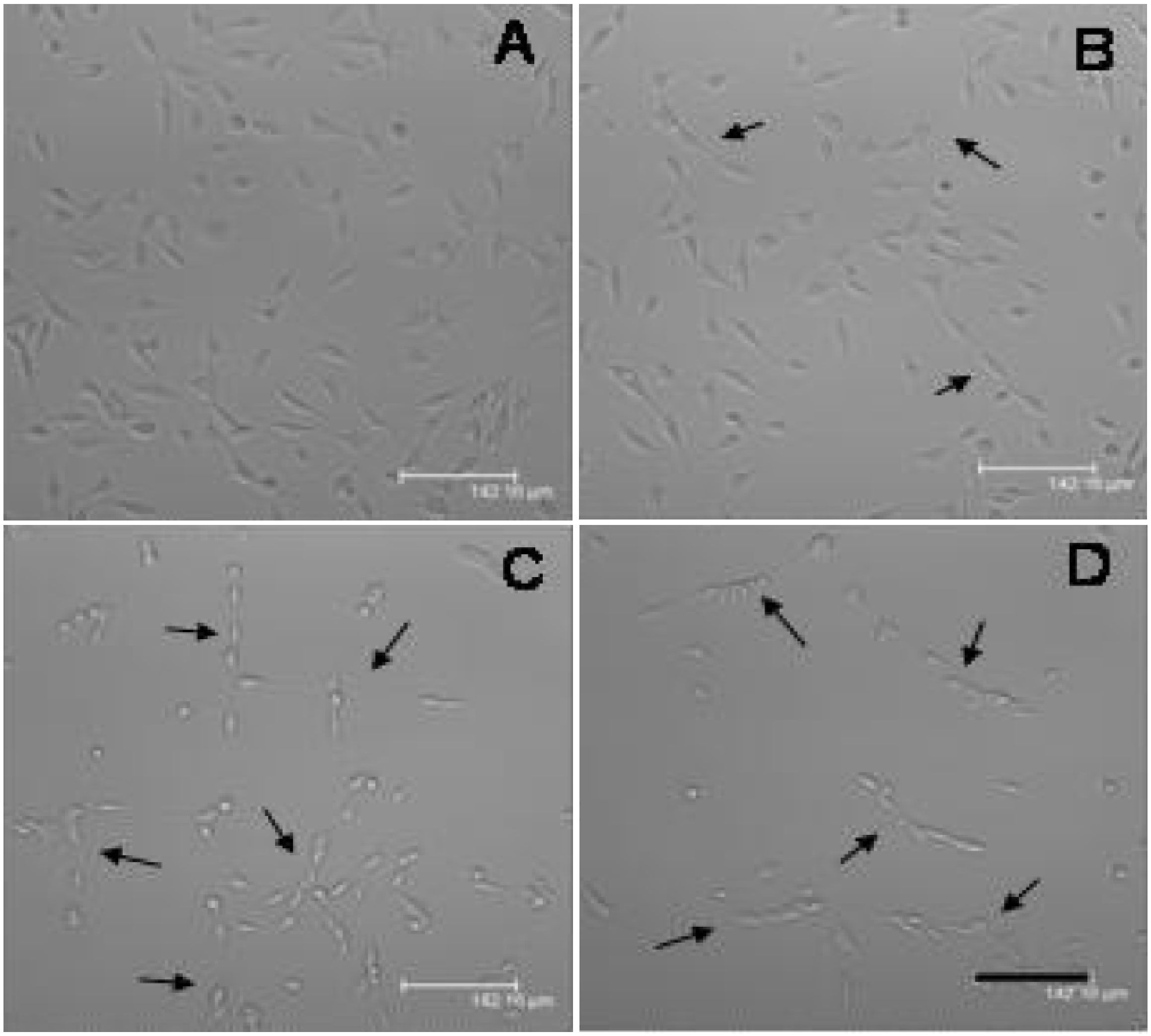

Additional File 1:

Cell clustering on nanopatterned Agrin functionalized substrates. Depicted are representative light microscopy images from B35 rat neuroblastoma cells on 30 nm (A), 60 nm (B), 90 nm (C), and 160 nm (D). The percentage of cells in clusters on 30 nm and 60 nm substrates are lower than 20 percent, However, on 90 nm and 160 nm more than 80 percent of the cells are clustered. Clustering is a dynamic process, on all substrates, cells are initially isolated and usually stay isolated from other cells for about three hours. Pictures were taken after 24 hours in culture.
