# Supplementary material for: Cell adhesion to agrin presented as a nanopatterned substrate is consistent with an interaction with the extracellular matrix and not transmembrane adhesion molecules
Source: BMC Cell Biol. 2008 Dec 4;9:64. doi: 10.1186/1471-2121-9-64 (PMC2612657; doi:10.1186/1471-2121-9-64)
Supplement: Additional file 2 — Cell spreading on nanopatterned substrates. Cells show greater spreading on closely spaced Agrin substrates (30, 60 nm), but have rounder morphologies with less surface area in contact with 90 and 160 nm substrates. [file 1471-2121-9-64-S2.pdf]

Additional file 2

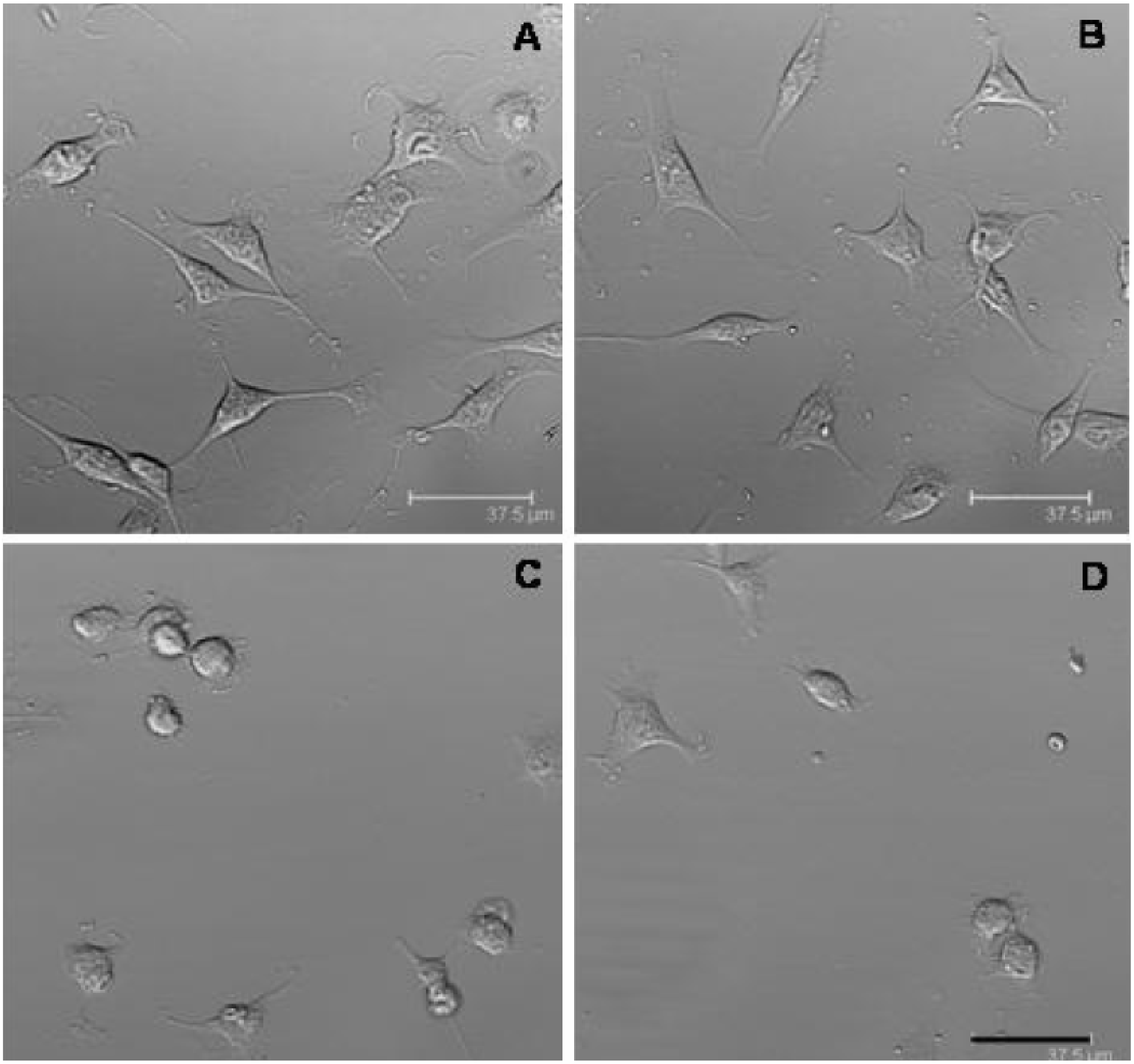

Graph 1

Spreading factor on different agrin nano-patterns.

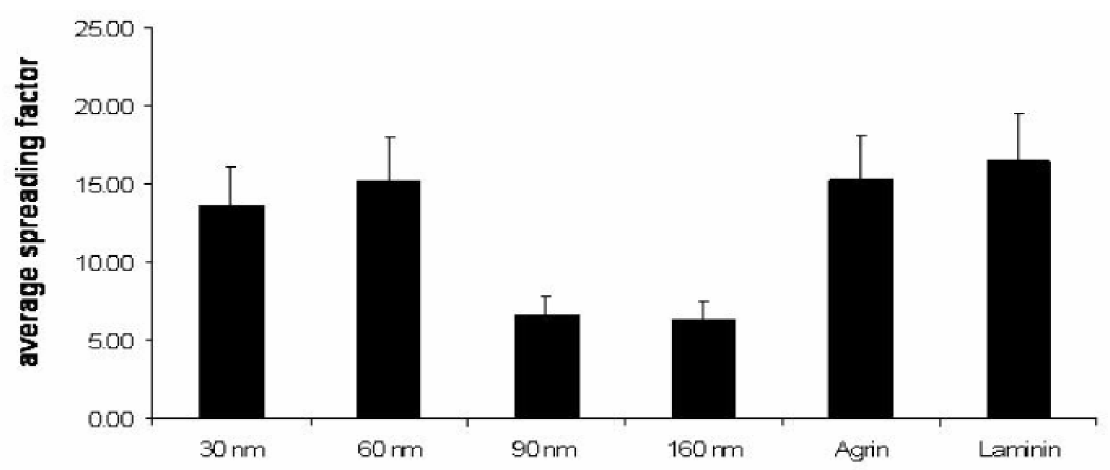

Additional File 2:

Cell spreading on nanopatterned substrates. B 35 rat neuroblastoma cells were imaged with DIC optics on 30 nm, 60 nm, 90 nm, and 160 nm (A-D) structured Agrin substrates four hours after plating. On 30 nm and 60 nm substrates, cells extend long protrusions resembling the morphology seen in Petri dish culture. On 90 nm and 160 nm substrates cells are more round, without large protrusions. On 30 and 60 nm substrates, the cell surface area (area covered by the cell soma) nearly double compared to the more widely spaced substrates. These results are quantified in the accompanying graph.
